# Supplementary material for: A computational predictor of the anaerobic mechanical power outputs from a clinical exercise stress test
Source: PLoS One. 2023 May 5;18(5):e0283630. doi: 10.1371/journal.pone.0283630 (PMC10162510; doi:10.1371/journal.pone.0283630)
Supplement: S1 Table — Calculated features obtained from a GXT. (PDF) [file pone.0283630.s001.pdf]

Table A in S1 File. Features description

GXT- based Feature	Description
Max HR	Based on the equation of Tanaka, H. et al.
100% HR max	100% of maximal, age-predicted HR
90% of max HR max	90% of maximal, age-predicted HR
85% of max HR max	85% of maximal age-predicted HR
VO_2 predicted at 100% of max HR (mL/min)	Calculating 100% of the predicted maximal oxygen consumption in absolute values using mL/min unit (which was also calculated for 90% and 85% of maximal age-predicted HR)
VO_2 predicted at 100% of max HR (mL/min/Kg)	Calculating 100% of the predicted maximal oxygen consumption in relative values using mL/min/kg unit (which was also calculated for 90% and 85% of maximal age-predicted HR)
Slope * speed at 100% of max HR	Maximal value of slope multiplied by the maximal value of speed (which was also calculated for 90% and 85% of maximal age-predicted HR)
Max intensity at 100% of max HR	Maximal value of slope multiplied by the maximal value of speed multiplied by the duration of the test at 100% of maximal age-predicted HR (which was also calculated for 90% and 85% of maximal age-predicted HR)
Exercise time at 100% of max HR	Time at 100% of maximal HR (which was also calculated for 90% and 85% of maximal age-predicted HR)
VO_2 at AT (mL/min) at 100% of max HR	Oxygen consumption at ventilatory anaerobic threshold based on the duration of the test , using mL/min unit (which was also calculated for 90% and 85% of maximal age-predicted HR)
VO_2 at AT (mL/min/kg) at 90% of max HR	Oxygen consumption at ventilatory anaerobic threshold based on the duration of the test using mL/min/kg unit (which was also calculated for 90% and 85% of maximal age-predicted HR)

Fig. S1. Calculated features obtained from a GXT.
