# Supplementary material for: A computational predictor of the anaerobic mechanical power outputs from a clinical exercise stress test
Source: PLoS One. 2023 May 5;18(5):e0283630. doi: 10.1371/journal.pone.0283630 (PMC10162510; doi:10.1371/journal.pone.0283630)
Supplement: S2 Table — (PDF) [file pone.0283630.s002.pdf]

Table B in S2 File. Data of GXT features

Age	Weight	Height	Age predicted HR max	85% of Age predicted HR max	90% of Age predicted HR max	VO2 predicted 85% of Age predicted HR max (mL/min/Kg)	VO2 predicted at 90% of Age predicted HR max(mL/min/Kg)	VO2 predicted at 100% of Age predicted HR max (mL/min/Kg)	Incline * speed at 85% of of Age predicted HR max	Incline * speed at 90% of Age predicted HR max	Incline * speed at 100% of Age predicted HR max	time at 85% of Age predicted HR max	time at 90% of of Age predicted HR max	time at 100% of of Age predicted HR max	Incline * speed *time at 85% of of Age predicted HR max	Incline * speed *time at 90% of Age predicted HR max	Incline * speed *time at 100% of of Age predicted HR max	VO2 at AT - 85% of Age predicted HR max	VO2 at AT - 90% of Age predicted HR max	VO2 at AT - 100% of Age predicted HR max	Predicted VO2 - 85% of Age predicted HR max(ml/min)	VO2 at AT (ml/min)- 85% of Age predicted HR max	Predicted VO2(ml/min)-90% of Age predicted HR max	VO2 at AT (ml/min)-90% of Age predicted HR max	Predicted VO2(ml/min)-100% of Age predicted HR max	VO2 at AT (ml/min)-100% of Age predicted HR max	Peak Power	Mean Power
23	72	174	192	163	173	50.7	50.7	66.3	26.0	10920	85800	345	420	660	8970	10920	85800	30.4	30.4	49.8	3653	2192	3653	2192	4776	3582	803.8	583.2
30	68	167	187	159	168	45.8	48.8	51.8	60.0	40800	61500	450	510	615	27000	40800	61500	27.5	34.2	38.9	3103	1862	3306	2314	3509	2632	768.4	528.1
27	72	172	189	161	170	50.7	58.5	66.3	26.0	45630	91650	495	585	705	12870	45630	91650	35.5	41.0	49.8	3643	2550	4203	2942	4763	3572	853.9	613.2
23	74	171	192	163	173	39.8	43.5	53.4	20.0	4290	36960	105	195	420	2100	4290	36960	21.9	23.9	32.0	2948	1621	3217	1769	3949	2369	687.2	497.9
34	66	164	184	157	166	36.2	39.8	47.3	18.0	6000	35700	240	300	510	4320	6000	35700	19.9	21.9	33.1	2375	1306	2613	1437	3105	2174	527.8	428.4
22	67	169	193	164	173	28.9	30.7	43.4	36.0	14400	44880	255	300	510	9180	14400	44880	15.9	16.9	30.4	1936	1065	2057	1131	2906	2034	477.2	344.4
22	58	163	193	164	173	36.2	38.9	47.0	18.0	15660	52650	345	435	585	6210	15660	52650	21.7	23.3	32.9	2100	1260	2256	1354	2726	1908	472.5	359.6
22	52	162	193	164	173	43.5	43.5	54.3	22.0	5940	34560	240	270	480	5280	5940	34560	23.9	23.9	46.2	2260	1243	2260	1243	2824	2400	357.3	259.2
26	67	170	190	161	171	39.8	42.8	42.8	20.0	17400	17400	270	435	435	5400	17400	17400	21.9	25.7	25.7	2669	1468	2870	1722	2870	1722	477.9	311.4
27	65	184	189	161	170	50.7	54.6	66.3	26.0	20280	76050	300	390	585	7800	20280	76050	27.9	32.8	46.4	3298	1814	3551	2131	4312	3018	775.2	576.0
22	52	162	193	164	173	35.0	35.0	39.8	32.0	10080	31680	270	315	495	8640	10080	31680	19.2	21.0	27.8	1818	1000	1818	1091	2068	1448	395.4	269.3
26	78	187	190	161	171	50.7	54.3	61.5	48.0	39960	81000	495	555	675	23760	39960	81000	35.5	38.0	46.1	3929	2750	4208	2946	4766	3575	833.5	673.1
27	59	163	189	161	170	46.8	53.4	56.7	44.0	50160	72600	480	570	660	21120	50160	72600	39.8	37.4	42.5	2741	2329	3127	2189	3321	2491	543.2	409.0
25	103	190	191	162	171	50.7	50.7	62.4	26.0	9750	54600	285	375	525	7410	9750	54600	27.9	30.4	43.7	5226	2874	5226	3135	6431	4501	1184.1	855.8
23	78	177	192	163	173	32.6	35.0	37.4	16.0	11520	22320	255	360	465	4080	11520	22320	17.9	21.0	22.4	2553	1404	2741	1645	2930	1758	508.7	370.8
23	71	178	192	163	173	47.1	47.1	57.9	24.0	10440	59040	330	435	615	7920	10440	59040	28.3	28.3	43.4	3349	2009	3349	2009	4117	3088	801.9	607.9
26	63	176	190	161	171	43.5	46.8	48.4	22.0	18480	27225	330	420	495	7260	18480	27225	26.1	28.1	33.9	2717	1630	2923	1754	3026	2118	626.7	478.7
25	54	168	191	162	171	39.8	43.5	50.1	20.0	7590	36630	285	345	555	5700	7590	36630	21.9	26.1	35.0	2151	1183	2347	1408	2704	1893	477.3	352.1
23	62	174	192	163	173	45.8	45.8	47.3	60.0	29700	38850	450	495	555	27000	29700	38850	27.5	32.1	33.1	2842	1705	2842	1989	2935	2054	427.5	344.8
23	50	169	192	163	173	39.8	39.8	45.8	20.0	5400	26100	210	270	435	4200	5400	26100	21.9	21.9	27.5	1992	1095	1992	1095	2292	1375	473.8	328.6
25	90	185	191	162	171	43.5	53.4	56.7	22.0	46200	69300	375	525	630	8250	46200	69300	26.1	37.4	42.5	3912	2347	4803	3362	5100	3825	975.7	772.7
28	53	152	188	160	170	39.8	45.8	50.3	20.0	25200	45900	300	420	510	6000	25200	45900	21.9	27.5	35.2	2123	1168	2443	1466	2683	1878	406.7	316.3
29	65	170	188	160	169	38.9	41.6	49.4	36.0	25920	68400	435	480	720	15660	25920	68400	23.3	35.4	42.0	2525	1515	2700	2295	3207	2726	618.5	452.4
26	47	156	190	161	171	39.8	39.8	47.3	20.0	8400	40950	330	420	585	6600	8400	40950	23.9	23.9	33.1	1852	1111	1852	1111	2201	1541	414.1	306.4
24	63	160	191	163	172	38.9	40.3	44.3	36.0	20925	47520	390	465	660	14040	20925	47520	23.3	24.2	33.2	2466	1480	2552	1531	2809	2106	473.4	362.6
25	64	167	191	162	171	54.4	54.4	67.0	28.0	8820	60480	270	315	540	7560	8820	60480	29.9	32.6	46.9	3458	1902	3458	2075	4259	2981	664.0	517.1
22	61	162	193	164	173	46.8	50.1	50.1	44.0	36630	36630	360	555	555	15840	36630	36630	28.1	35.0	35.0	2853	1712	3054	2138	3054	2138	525.0	416.1
26	50	162	190	161	171	36.2	36.2	41.6	18.0	6750	29970	315	375	555	5670	6750	29970	21.7	21.7	29.1	1810	1086	1810	1086	2080	1456	296.3	213.0
23	64	169	192	163	173	36.2	38.9	44.3	18.0	15660	38880	345	435	540	6210	15660	38880	21.7	23.3	31.0	2317	1390	2490	1494	2835	1985	442.2	329.4
30	86	185	187	159	168	47.1	50.7	62.4	24.0	23760	70200	375	495	675	9000	23760	70200	28.3	35.5	46.8	4065	2439	4375	3063	5388	4041	892.6	697.8
21	64	166	193	164	174	36.2	41.6	47.3	18.0	21060	38850	315	390	555	5670	21060	38850	21.7	25.0	33.1	2328	1397	2675	1605	3044	2130	507.1	372.9
21	95	185	193	164	174	47.1	54.3	57.9	24.0	37800	56160	435	525	585	10440	37800	56160	28.3	38.0	40.5	4460	2676	5142	3600	5483	3838	852.6	678.1
24	56	164	191	163	172	52.5	52.5	52.5	60.0	33300	33300	525	555	555	31500	33300	33300	36.8	36.8	36.8	2940	2058	2940	2058	2940	2058	367.0	279.6
23	65	177	192	163	173	43.5	53.4	55.0	22.0	44880	59400	375	510	600	8250	44880	59400	26.1	37.4	46.8	2838	1703	3485	2439	3593	3054	614.9	513.3
29	76	177	188	160	169	54.3	57.9	63.3	72.0	37440	73260	330	390	555	23760	37440	73260	32.6	34.7	44.3	4127	2476	4400	2640	4811	3368	851.6	653.7
26	81	175	190	161	171	47.3	48.8	48.8	70.0	42000	42000	480	525	525	33600	42000	42000	40.2	34.2	34.2	3810	3239	3931	2752	3931	2752	953.9	714.8
29	67	185	188	160	169	47.1	54.3	67.0	24.0	29160	52650	255	405	585	6120	29160	52650	25.9	32.6	46.9	3132	1723	3611	2167	4456	3119	635.9	517.3
23	59	161	192	163	173	37.4	41.6	44.3	48.0	21060	34560	330	390	480	15840	21060	34560	22.4	25.0	37.7	2220	1332	2471	1483	2631	2237	522.7	353.4
23	60	151	192	163	173	32.6	36.2	41.6	16.0	5130	25920	225	285	480	3600	5130	25920	17.9	19.9	35.4	1954	1075	2172	1195	2496	2122	394.1	304.2
23	61	165	192	163	173	36.2	39.8	42.8	18.0	6000	17400	240	300	435	4320	6000	17400	19.9	21.9	25.7	2208	1215	2430	1336	2613	1568	308.4	221.3
23	53	154	192	163	173	31.4	34.9	38.9	8.0	2160	17280	195	240	480	1560	2160	17280	17.3	19.2	33.1	1662	914	1847	1016	2062	1752	363.6	265.1
26	88	176	190	161	171	43.5	46.8	50.1	22.0	15180	32670	285	345	495	6270	15180	32670	23.9	28.1	35.0	3825	2104	4115	2469	4406	3084	780.8	571.9
23	60	172	192	163	173	48.4	48.4	48.4	55.0	19800	19800	360	360	360	19800	19800	19800	29.1	29.1	29.1	2905	1743	2905	1743	2905	1743	430.2	332.7
37	95	178	182	155	164	45.8	50.1	53.4	60.0	31680	54120	420	480	615	25200	31680	54120	27.5	42.6	40.0	4359	2615	4761	4047	5075	3806	929.4	702.8
26	70	170	190	161	171	45.8	45.8	47.3	60.0	22500	30450	345	375	435	20700	22500	30450	27.5	27.5	28.4	3208	1925	3208	1925	3313	1988	440.5	313.2
24	78	175	191	163	172	38.9	44.3	51.8	36.0	36720	66000	420	510	660	15120	36720	66000	23.3	31.0	38.9	3038	1823	3460	2422	4048	3036	753.8	588.9
27	60	173	189	161	170	38.9	41.6	50.1	36.0	26730	38610	375	495	585	13500	26730	38610	23.3	29.1	35.0	2334	1400	2496	1747	3004	2103	429.9	345.2
29	67	185	188	160	169	47.1	54.3	67.0	24.0	29160	52650	240	405	585	5760	29160	52650	25.9	32.6	46.9	3132	1723	3611	2167	4456	3119	635.9	521.4
21	66	176	193	164	174	67.0	69.3	69.3	90.0	64575	64575	510	615	615	45900	64575	64575	46.9	51.9	51.9	4422	3095	4571	3428	4571	3428	708.2	540.7
33	62	159	185	157	166	36.2	38.9	44.3	18.0	14580	41040	315	405	570	5670	14580	41040	21.7	23.3	31.0	2244	1347	2412	1447	2747	1923	481.5	356.5
28	78	177	188	160	170	43.5	43.5	53.4	22.0	6600	44880	270	300	510	5940	6600	44880	23.9	23.9	37.4	3390	1865	3390	1865	4163	2914	796.5	620.9
26	66	164	190	161	171	43.5	47.1	52.5	22.0	7920	29700	240	330	495	5280	7920	29700	23.9	28.3	36.8	2869	1578	3109	1865	3465	2426	627.8	441.7
23	57	167	192	163	173	39.8	39.8	42.8	20.0	7200	21000	300	360	525	6000	7200	21000	21.9	23.9	30.0	2271	1249	2271	1362	2442	1709	312.4	211.6
26	77	179	190	161	171	45.8	48.8	57.8	60.0	35120	88620	379	439	633	22740	35120	88620	27.5	29.3	43.4	3529	2118	3760	2256	4453	3340	774.5	624.0
25	69	169	191	162	171	40.2	43.5	58.6	11.0	3204	27496	203	267	491	2233	3204	27496	22.1	23.9	41.0	2784	1531	3015	1658	4059	2841	724.2	524.0
27	80	171	189	161	170	46.8	50.2	67.0	13.0	6636	61290	407	474	681	5291	6636	61290	28.1	30.1	50.3	3737	2242	4003	2402	5347	4010	915.7	729.7
31	76	183	186	158	168	51.8	60.5	69.2	14.5	33640	83636	429	580	721	6221	33640	83636	31.1	42.4	55.4	3913	2348	4570	3199	5227	4182	738.6	609.1
27	93	177	189	161	170	36.8	40.2	50.7	10.0	3091	21648	210	281	451	2100	3091	21648	20.3	22.1	30.4	3422	1882	3731	2052	4710	2826	987.8	703.9
32	77	182	186	158	167	43.5	46.8	67.0	12.0	4108	70336	252	316	628	3024	4108	70336	23.9	28.1	50.2	3367	1852	3625	2175	5183	3887	813.2	623.0
29	61	170	188	160	169	53.5	63.5	76.3	15.0	6516	43216	220	362	584	3300	6516	43216	29.4	38.1	53.4	3269	1798	3880	2328	4660	3262	623.7	446.9
30	73	170	187	159	168	30.2	33.5	42.8	8.0	1449	13160	81	161	329	648	1449	13160	16.6	18.4	25.7	2214	1218	2459	1352	3144	1886	589.2	407.0
24	81	186	191	163	172	54.4	58.6	67.0	28.0	31304	79520	496	559	710	13888	31304	79520	38.1	41.0	50.2	4387	3071	4726	3308	5404	4053	796.9	517.5
35	57	173	184	156	165	43.5	46.8	53.4	22.0	14124	38016	279	321	432	6138	14124	38016	23.9	28.1	32.0	2465	1356	2652	1591	3026	1816	541.7	408.0
24	63	171	191	163	172	43.5	50.7	65.1	12.0	19248	90432	285	401	628	3420	19248	90432	23.9	30.4	48.8	2749	1512	3204	1923	4114	3086	625.6	426.3
23	59	158	192	163	173	41.6	44.3	47.0	54.0	23688	33750	244	329	375	13176	23688	33750	22.9	26.6	28.2	2463	1354	2623	1574	2782	1669	376.2	270.2
21	60	157	193	164	174	40.2	40.2	57.9	11.0	2882	53952	247	262	562	2717	2882	53952	22.1	22.1	40.5	2402	1321	2402	1321	3462	2424	665.8	512.8
34	80	179	184	157	166	40.2	43.5	58.6	11.0	3852	32592	255	321	582	2805	3852	32592	22.1	26.1	41.0	3213	1767	3480	2088	4685	3280	844.8	646.2
29	75	179	188	160	169	44.8	48.0	57.4	42.0	25767	68796	345	409	546	14490	25767	68796	26.9	28.8	40.2	3373	2024	3611	2166	4322	3026	812.5	589.2
22	65	166	193	164	173	40.2	43.5	54.6	11.0	2856	23504	179	238	452	1969	2856	23504	22.1	23.9	32.8	2615	1438	2832	1558	3557	2134	474.0	376.3
36	86	187	183	155	165	46.8	50.2	61.6	13.0	6412	20928	368	458	654	4784	6412	20928	28.1	30.1	46.2	4042	2425	4329	2598	5319	3989	898.0	703.1
36	80	182	183	155	165	40.2	43.5	65.1	11.0	3588	97920	249	299	680	2739	3588	97920	22.1	23.9	48.8	3217	1770	3484	1916	5215	3911	851.3	636.8

Age	Weight	Height	Age predicted HR max	85% of Age predicted HR max	90% of Age predicted HR max	VO2 predicted 85% of Age predicted HR max (mL/min/Kg)	VO2 predicted at 90% of Age predicted HR max(mL/min/Kg)	VO2 predicted at 100% of Age predicted HR max (mL/min/Kg)	Incline * speed at 85% of Age predicted HR max	Incline * speed at 90% of Age predicted HR max	Incline * speed at 100% of Age predicted HR max	time at 85% of Age predicted HR max	time at 90% of of Age predicted HR max	time at 100% of of Age predicted HR max	Incline * speed *time at 85% of of Age predicted HR max	Incline * speed *time at 90% of Age predicted HR max	Incline * speed *time at 100% of Age predicted HR max	VO2 at AT - 85% of Age predicted HR max	VO2 at AT - 90% of Age predicted HR max	VO2 at AT - 100% of Age predicted HR max	Predicted VO2 - 85% of Age predicted HR max(ml/min)	VO2 at AT (ml/min)-85% of Age predicted HR max	Predicted VO2(ml/min)-90% of Age predicted HR max	VO2 at AT (ml/min)-90% of Age predicted HR max	Predicted VO2(ml/min)-100% of Age predicted HR max	VO2 at AT (ml/min)-100% of Age predicted HR max	Peak Power	Mean Power
26	55	159	190	161	171	36.8	40.2	48.7	10.0	2651	19320	188	241	420	1880	2651	19320	20.3	22.1	29.2	2026	1114	2209	1215	2680	1608	437.5	323.8
34	76	179	184	157	166	38.5	41.7	57.4	10.5	7707	72954	323	367	579	3392	7707	72954	23.1	25.0	40.2	2907	1744	3145	1887	4334	3034	670.0	474.4
33	61	155	185	157	166	54.6	54.6	54.6	52.0	25636	25636	493	493	493	25636	25636	25636	38.2	38.2	38.2	3338	2337	3338	2337	3338	2337	398.7	298.4
30	83	174	187	159	168	33.5	46.8	62.8	9.0	4719	49896	177	363	594	1593	4719	49896	18.4	28.1	43.9	2770	1524	3873	2324	5191	3634	561.3	397.3
50	94	177	173	147	156	35.2	38.0	43.7	9.5	5016	23484	180	264	412	1710	5016	23484	19.3	20.9	26.2	3313	1822	3581	1970	4118	2471	842.6	542.7
40	85	182	180	153	162	40.2	46.8	58.5	11.0	3952	40404	197	304	518	2167	3952	40404	22.1	28.1	41.0	3406	1873	3971	2383	4964	3475	823.2	603.8
37	76	172	182	155	164	43.5	46.8	58.5	12.0	4485	40248	287	345	516	3444	4485	40248	23.9	28.1	41.0	3315	1823	3569	2141	4460	3122	779.3	555.1
26	65	174	190	161	171	47.1	54.3	68.7	24.0	31104	112728	363	432	671	8712	31104	112728	28.3	32.6	51.5	3066	1840	3535	2121	4472	3354	694.7	550.0
22	68	165	193	164	173	43.5	50.7	61.5	12.0	17952	68160	273	374	568	3276	17952	68160	23.9	30.4	43.1	2962	1629	3453	2072	4188	2932	694.8	495.0
36	88	177	183	155	165	40.2	46.8	58.6	11.0	4875	33376	264	375	596	2904	4875	33376	22.1	28.1	41.0	3523	1937	4107	2464	5136	3595	809.3	589.5
30	56	163	187	159	168	45.3	52.2	52.2	23.0	36915	36915	364	535	535	8372	36915	36915	27.2	36.5	36.5	2540	1524	2927	2049	2927	2049	459.7	370.6
28	78	181	188	160	170	68.9	79.7	79.7	36.0	86184	86184	672	798	798	24192	86184	86184	51.7	63.8	63.8	5360	4020	6201	4961	6201	4961	731.7	466.4
39	74	180	181	154	163	56.8	66.4	76.0	16.0	37056	94848	518	579	741	8288	37056	94848	39.8	46.5	60.8	4183	2928	4889	3423	5596	4477	801.4	607.1
22	73	180	193	164	173	50.2	54.4	67.0	14.0	14588	80528	439	521	719	6146	14588	80528	30.1	38.1	50.2	3657	2194	3963	2774	4882	3661	801.4	592.7
32	87	172	186	158	167	36.8	42.8	42.8	10.0	12880	14520	198	322	363	1980	12880	14520	20.3	25.7	25.7	3193	1756	3714	2228	3714	2228	721.6	450.8
33	86	166	185	157	166	26.8	32.6	37.4	7.0	1120	10272	3	70	214	21	1120	10272	14.8	17.9	20.6	2302	1266	2794	1537	3206	1763	547.9	416.8
24	55	161	191	163	172	48.5	60.7	60.7	13.5	59076	59076	569	729	729	7685	59076	59076	34.0	48.5	48.5	2668	1867	3336	2669	3336	2669	406.5	312.9
25	53	161	191	162	171	36.8	43.5	50.7	10.0	3048	19632	129	254	409	1290	3048	19632	20.3	23.9	30.4	1945	1070	2297	1263	2677	1606	390.4	319.7
36	91	165	183	155	165	26.8	26.8	33.1	7.0	392	9114	14	56	217	98	392	9114	14.8	14.8	18.2	2442	1343	2442	1343	3015	1658	666.0	310.0
28	53	152	188	160	170	33.5	42.8	48.8	9.0	14600	44800	235	365	560	2115	14600	44800	18.4	25.7	34.2	1782	980	2279	1367	2598	1819	501.5	366.9
34	52	162	184	157	166	33.5	38.9	44.3	9.0	9072	27216	147	252	378	1323	9072	27216	18.4	21.4	26.6	1752	964	2034	1119	2317	1390	347.4	214.8
32	73	178	186	158	167	50.2	56.8	75.5	14.0	7696	75582	362	481	741	5068	7696	75582	30.1	39.8	60.4	3652	2191	4137	2896	5494	4395	817.3	632.0
