# Supplementary material for: A computational predictor of the anaerobic mechanical power outputs from a clinical exercise stress test
Source: PLoS One. 2023 May 5;18(5):e0283630. doi: 10.1371/journal.pone.0283630 (PMC10162510; doi:10.1371/journal.pone.0283630)
Supplement: Attachment — Submitted filename: Leopold Reply letter to reviewers Final.docx [file pone.0283630.s003.docx]

May 24^th^, 2022

Juan M. Murias

Editor-in-Chief

*PLOS ONE*

Dear Editor-in-Chief,

We have substantially revised our manuscript "**A computational predictor of the anaerobic mechanical power outputs from a clinical exercise stress test**” following the reviewer’s comments. We are sorry for the delay in our reply but believe that the current version of our manuscript meets the criteria of PLoS One Journal.

In the following, we referred to each of the reviewer’s comments, and referred our corrections to the specific lines in the manuscript.

Sincerely yours,

Prof. Mickey Scheinowitz	Prof Tamir Tuller

Reviewer #1:

In this study, the authors attempt to determine whether variables collected during a graded exercise test to exhaustion without concurrent gas exchange and ventilatory measures could be used to predict peak and mean power output achieved during a 30-s Wingate test. Participants performed on two separate occasions, a 30-s Wingate on a cycle ergometer and a graded exercise test on a treadmill. Metrics derived from the graded exercise test combined with participant characteristics were acquired and then applied to a multiple linear regression analysis that identified predictor variables of statistical significance. The accuracy of the model was then tested in a subset of participants (which [I think] were not used to build the original model) and the model demonstrated a modest degree of accuracy in predicting peak and mean cycling power.

Major Issue:

1. I found the description of the study methodology nearly impossible to follow and lacking sufficient detail for replication.

We agree with the reviewer that the description of the methodology was confusing, we therefore substantially revised the entire manuscript including the Methods section.

2. In particular, the rationale, process, and relevance of the 85%, 90%, and 100% of age-predicted HR max analysis is not clear at all. Why was this done? How was it done?

The aim of our study was to predict the anaerobic mechanical power outputs from (1) a clinical ergometry with no gas exchange, and (2) maximal as well as sub-maximal stress test. To do that we used data that was obtained from our lab during cardiopulmonary exercise testing in which the test was terminated when the subject reached her/his VO_2_max level.

In the current manuscript we used the following methodology:

(1) We took the subject's **age** and calculated heart rate (HR) max (100%) based on Tanaka H et al. [1] formula, which equal to 208 - 0.7 x age.

(2) We then took the calculated HRmax value and superimposed that value with the treadmill's speed and slope obtained from the cardiopulmonary exercise testing. Those data served us to calculate **VO_2_max** based on ACSM'S metabolic calculations handbook: VO2max= (0.2 · Speed) + (0.9 · Speed · Grade) + 3.5

(3) Since “clinical ergometry” usually ends when the subject reaches 85% to 90% of his/her HRmax [2], we calculated the 90% and the 85% HRmax values to reflect submaximal HR values. Those calculated values were used as features to predict the anaerobic mechanical power output.

We found that the best prediction correlation for peak anaerobic mechanical power output (PP) and mean mechanical power output (MP) are 0.93 and 0.94, respectively. This means that in practice for peak power, a sub-maximal (85% of HRmax) value is sufficient, while maximal value (100% of HRmax) is required to best predict the mean power output.

[1] Tanaka H, Monahan KD, Seals DR. Age-predicted maximal heart rate revisited. J Am Coll Cardiol. 2001 Jan;37(1):153-6.

[2] Noonan, Vanessa, and Elizabeth Dean. "Submaximal exercise testing: clinical application and interpretation." Physical therapy 80.8 (2000): 782-807.

3. I cannot discern the answer to these important questions with the manuscript in its present form. For this reason, I am unable to judge the validity of the methods, results and to determine whether the authors’ interpretation and conclusions are consistent with these.

We hope that the substantial revision made in the background and more widely in the method section is sufficient to better understand the rational of the study and its methodology used.

Other Comments:

4. The explanation regarding anaerobic contributions to incremental exercise is difficult to follow. What do the authors mean by “anaerobic threshold”? This term has been used to describe both the lactate threshold (i.e., metabolic rate at which blood lactate first becomes elevated above resting concentrations; often referred to as LT1) and the critical power/intensity (i.e., metabolic rate at which blood lactate accumulation accelerates; often referred to as LT2).

We are aware that there are different approaches to detect the anaerobic threshold [1], yet in this paper we treat the data as clinical data, without knowing the VO_2_, and therefore LT1 and LT2 are irrelevant in this paper. We have only used calculated VO_2_ through the ACSM equation as mentioned in the methods sections, lines 52-54.

Since anaerobic threshold is not measured during a clinical exercise stress test (not ventilatory nor blood lactate), we estimated the exercise intensity for training reflected as target training heart rate [2], which is equivalent to the anaerobic threshold. The estimated training heart rate should be around the anaerobic threshold [3] so it will induce improvement in exercise capacity. That level should be just before the individual becomes acidosis (starting to use the anaerobic metabolism).

In the introduction we have mentioned the anaerobic threshold to explained the rationale behind the paper which is that above the anaerobic threshold (AT) there is additional usage of the anaerobic metabolic pathways and we would like to find it. However, we agree with the reviewer comment and changed this sentence in the introduction lines 31-35-

“In this paper we aim to predict the anaerobic mechanical power outputs from a grade exercise test (GXT), which has no gas exchange measurements. Our assumption was that during GXT, the subject reaches maximal or submaximal intensity, and at this point, anaerobic metabolism becomes dominate and allows the generation of additional mechanical power output”.

[1] [Keir, Daniel A., et al. "Identification of Non-Invasive Exercise Thresholds: Methods, Strategies, and an Online App." *Sports Medicine* (2021): 1-19.

[2] Lear, Scott A., et al. "Exercise stress testing." Sports medicine 27.5 (1999): 285-312.‏

[3] Ghosh AK. Anaerobic threshold: its concept and role in endurance sport. Malays J Med Sci. 2004;11(1):24-36.

5. Why was the graded exercise test performed on a treadmill instead of a cycling ergometer. Do the author’s think it is justifiable to use treadmill exercise to predict cycling performance?

The Wingate test is performed on cycle ergometer whereas clinical aerobic test is usually performed on treadmill. We are aware that there is a difference between cycle and treadmill ergometer and the use of both of them is not ideal, yet in the current manuscript we have managed to show high prediction correlation of the anaerobic mechanical power outputs using a running test. Actually, since the use of both cycle and treadmill ergometer is not ideal, we would expect higher correlation when using the same ergometer. Therefore, we might report a lower correlation on this paper since the real performances might even be higher.

In addition, in a previous study [1] we have shown high correlations between the anaerobic mechanical power outputs performed on a cycle ergometer and a maximal incremental cardiopulmonary test during running (with gas exchange measurements). Therefore, we thought to explore, in the current study, whether these data will repeat itself in a regular exercise stress test, namely, clinical ergometry.

[1] Leopold E, Navot-Mintzer D, Shargal E, Tsuk S, Tuller T, Scheinowitz M. Prediction of the Wingate anaerobic mechanical power outputs from a maximal incremental cardiopulmonary exercise stress test using machine-learning approach. PLoS One

6. From a clinical standpoint, why is would it be relevant to quantify the anaerobic capacity/power of a patient with chronic cardiovascular or respiratory disease?

In this paper, quantifying the anaerobic mechanical power output is mostly useful for exercise physiologist, coaches, etc. and not for patients including those with chronic cardiovascular or respiratory diseases. The idea of this paper was to understand the physiology involved with anaerobic mechanical power output as measured by peak power and mean power outputs. In the future, we would like to expand the model to patients with chronic cardiovascular or respiratory diseases.

7. Line 3: “EST” is not an abbreviation of “grade exercise test”. Consider GXT, which is commonly used.

We have changed the abbreviation to GXT along the manuscript.

8. Line 31: “Our assumption was that during EST….., and above it.” This is a crucial sentence to help explain the rationale. However, I do not know what the authors mean by it. Are they suggesting that once the critical power is surpassed during incremental exercise, anaerobic metabolism contributes increasingly to the power output and its capacity determines peak power output achieved?

Yes, this is exactly what we meant. The anaerobic metabolism contributes to the power output and its capacity during graded exercise test, and therefore our goal was to predict these mechanical power (peak (phosphagen-based) and mean (glycogen based)) power outputs.

9. Line 21: Please revise this sentence.

We have revised the sentence accordingly.

10. Line 42: “features”? why not variables or indices?

This is a more common word being used to describe variable when using models such as the one used in our paper [1-3].

[1] Chen, Rung-Ching, et al. "Selecting critical features for data classification based on machine learning methods." Journal of Big Data 7.1 (2020): 1-26.

[2] Silva, Thiago Christiano, and Liang Zhao. Machine learning in complex networks. Vol. 1. Cham, Switzerland: Springer International Publishing, 2016.‏

[3] Daumé, Hal. A course in machine learning. Hal Daumé III, 2017.‏

11. Line 44: “MP” not previously defined.

We have added the definition for mean power.

12. Line 46: This is unclear. Are the authors stating that the data from the previous study (i.e., [6]) were used? Or that the same variables as used to build the regression in [6] were used? This must be made clear.

In the current manuscript we have used the age, and treadmill speed and slope as obtained from our previous study. These variables were used to calculate the set of features described lengthy in the paper.

More specially, we took the subject's **age** and calculated heart rate (HR) max (100%) based on Tanaka H et al. [1] formula, which equal to 208 - 0.7 x age.

We then took the calculated HRmax value and superimposed that value with the treadmill's speed and slope obtained from the cardiopulmonary exercise testing. Those data served us to calculate **VO_2_max** based on ACSM'S metabolic calculations handbook: VO2max= (0.2 · Speed) + (0.9 · Speed · Grade) + 3.5

Since “clinical ergometry” usually ends when the subject reaches 85% to 90% of his/her HRmax [2], we calculated the 90% and the 85% HRmax values to reflect submaximal HR values. Those calculated values were used as features to predict the anaerobic mechanical power output.

[1] Tanaka H, Monahan KD, Seals DR. Age-predicted maximal heart rate revisited. J Am Coll Cardiol. 2001 Jan;37(1):153-6.

[2] Noonan, Vanessa, and Elizabeth Dean. "Submaximal exercise testing: clinical application and interpretation." Physical therapy 80.8 (2000): 782-807.

In order to make it clear we have further revised the method section in lines 46-59.

13. Lines 46-50: I am very confused here. What is meant by “CPET data was adjusted”? In what way? How?

Explanations made in the last comment.

We have provided more details in the Method section in lines 46-59.

14. Line 66: “first few seconds” – what constitutes a “few”? Please provide the exact time interval over which this was determined. Or, was PP simply the highest PO achieved at 1 second time intervals?

We have added this information to the main text (lines 74-75):

“PP output which was defined as the maximal mechanical power output achieved during the first few seconds of the test [1-5 sec]“

15. Line 73: 1.00 instead of 1.

We have changed it accordingly.

16. Line 74: Please provide information about the system used to acquire gas exchange and ventilatory variables.

We have added such information (lines 82-84):

“During the entire test, both the ventilatory and metabolic variables (such as VCO2 and VE) were recorded for each breath, using the Quark CPET (COSMED, Rome, Italy) until the subjects reached maximal effort.”

17. Line 93-94: Was the equation for predicting VO2max adjusted for age, sex, body mass, etc.? This should specified.

The VO2max equation was based on the ACSM'S metabolic calculations handbook:

VO2max= (0.2 · Speed) + (0.9 · Speed · Grade) + 3.5

Reviewer #2:

Leopold and colleagues aimed to investigate whether they could predict the anaerobic mechanical power output derived from an incremental test (submaximal and maximal) without gas exchange measures. The authors conducted a CPET and a Wingate test, and performed a linear regression to predict the anaerobic power output.

A few key points to consider:

1. After reading the introduction, I am still not sure what the authors mean by “anaerobic mechanical power outputs”.

The anaerobic mechanical power output is the peak power and mean power extracted from the Wingate test [1].

[1] Castañeda-Babarro, Arkaitz. "The Wingate Anaerobic Test, a Narrative Review of the Protocol Variables That Affect the Results Obtained." Applied Sciences 11.16 (2021): 7417.‏

Additionally, the use of the term “anaerobic threshold” should be avoided, as it is conceptually wrong. Please, refer to exactly what you mean, which I assume is the transition from the heavy to the severe-intensity domains.

We agree with the reviewer and therefore removed the term anaerobic threshold from the introduction and revised the sentence:

“In this paper we aim to predict the anaerobic mechanical power outputs from a GXT (clinical ergometry), which has no gas exchange measurements. Our assumption was that during GXT, the subject reaches maximal or submaximal exercise, and therefore requires to use anaerobic metabolism to generate additional mechanical power output.”

In this paper, we only used the term anaerobic threshold as one of the calculated features which is based on the subject's duration of the GXT. This is explained in details, in the supplement.

2. The authors mentioned “anaerobic components” throughout the manuscript. It was very unclear to me what this refers to exactly.

The Wingate test measures the anaerobic mechanical power outputs; peak power and mean power outputs. These outputs were predicted using variables taken from clinical GXT.

We emphasize in the introduction, lines 15-19, that the anaerobic components refer to the Mechanical Power Output by means of the Peak and Mean Power outputs.

3. The paper has many grammar mistakes and I suggest the authors to carefully revise the manuscript again.

We carefully revised the paper and corrected all grammar mistakes accordingly.

4. The authors aimed to compare results from a Wingate test with a CPET – they state that “the main observation of our current study is the ability to predict peak and mean anaerobic mechanical power outputs using a standard EST. Due to the high prediction model and outcomes, gas exchange and VO2 measurement is probably not necessary.”. The question is now: how can we derive “peak and mean anaerobic mechanical power outputs” from a CPET?

In our previous paper [1] we designed a computational model that included CPET variables (features) to predict the anaerobic mechanical power outputs from aerobic exercise stress test. In the current paper we used GXT features (which does not include gas exchange) to predict the anaerobic mechanical power outputs, since GXT is a more common test than CPET. We received high prediction correlations with minimal features (e.g. HR, treadmill time, speed and incline) even when calculated for sub-maximal test.

[1] Leopold E, Navot-Mintzer D, Shargal E, Tsuk S, Tuller T, Scheinowitz M. Prediction of the Wingate anaerobic mechanical power outputs from a maximal incremental cardiopulmonary exercise stress test using machine-learning approach. PLoS One

Given the above points, I believe the manuscript is conceptually flawed, and it does not add any value to the current literature.

We hope that by better explanation of the rational, the methodology used, and the unique results, will emphasize the contribution and novelty of this manuscript which is worthy for publication.

5. Specific comments:
   Li 11-12: this sentence does not make much sense to me. Please, specify what you mean by “anaerobic components”.

We emphasized in the Introduction, lines 15-19, that the anaerobic components refer to the Mechanical Power Output by means of Peak and Mean Power outputs as measured by the Wingate test. In other words, the anaerobic components refer to the anaerobic metabolism which add to the mechanical power output during the higher stages of an aerobic exercise stress test.

Li 21-22: please, revise this sentence.

We have revised this sentence:

“This information is of special interest for athletes [7-9], exercise physiologist and coaches who include anaerobic exercise in their training routine.”

Li 24: please, use the correct notation for VO2, with the dot above the V, and the subscript 2.

It shouldn’t be with a dot. We have revised to subscript 2.

Li 27: and where is the reference to Samantha et al?

I added this reference.

Li 29: please, remove this acronym (PP).

We have removed this acronym.

Li 31: you are missing a comma after EST.

We have fixed it.

Li 32-33: this sentence was a mix of concepts thrown altogether into two lines of text. What exactly do you mean by anaerobic threshold? And OBLA? And above it?

We have mentioned the anaerobic threshold in the Introduction section, in order to explain the rationale behind our hypothesis. The idea is that exercise level above the anaerobic threshold (AT) during GXT, uses anaerobic metabolic pathways which allows to generate additional mechanical power output.

As we mentioned in previous comment, we removed the term anaerobic thresholds and revised this sentence.

Li 35-36: I still do not know what anaerobic components are.

Please see the comment above.

Li 42: 51 features? Besides the fact that I have absolutely no idea what this refers to, this sounds like you threw everything inside a bucket hoping to find something in there.

The term feature is used to describe variable that is being used in models such as the one describe in our manuscript [1]. In this paper, we have used features that we believe can predict the anaerobic mechanical power outputs.

Machine learning is a method of data analysis that provides computers with the capability to learn without being explicitly programmed. When comparing to learning of human, machine learning is a faster method which can save and preserve more information, hence any progress in the field of machine learning, will have a strong influence on society [2]. The process of machine learning is a system which is using algorithms that iteratively learn from data, look for patterns, and allows computers to find hidden insights without being explicitly programmed where to look. The iterative aspect of machine learning is very important since as models are exposed to new data, they can teach themselves to grow and change and are able to independently adapt. They learn from previous computations to produce reliable, repeatable decisions and results.

In this paper, we developed a greedy heuristic algorithm to study the ability of a clinical aerobic GXT features to predict the anaerobic mechanical power outputs. This algorithm uses a locally optimal choice of features at each iteration. The algorithm automatically selects the ‘best’ feature (i.e. the feature that demonstrates the smallest distance between the predicted to the observed values of the anaerobic mechanical power output; see below) at each iteration and added it to the vector of selected features. The algorithm output is a linear regression mathematical equation that represents the best-chosen features, together with the features’ coefficients, which is also referred as the predictor.

[1] Mooney, Stephen J., and Vikas Pejaver. "Big data in public health: terminology, machine learning, and privacy." Annual review of public health 39 (2018): 95-112.

[2] Wang, H. et al. A Brief Review of Machine Learning and Its Application. International Conference on Information Engineering and Computer Science., Wuhan, China, pp. 1-4, 2009.

Li 44: What is PP and MP? It is somewhat cumbersome for the reader to come back to the acronym definition. My golden rule is that, if you were to say this word, would you use the acronym? If the answer is no, then you shouldn’t use an acronym for writing either.

It is very familiar acronym that being used widely in the literature: PP stands for Peak Power and MP stands for Mean Power. See references 1-2 for more information.

[1] Jaafar, Hamdi, et al. "Effects of load on Wingate test performances and reliability." The Journal of Strength & Conditioning Research 28.12 (2014): 3462-3468.‏

[2] Kohler, Ryan M., et al. "Peak power during repeated Wingate trials: implications for testing." The Journal of Strength & Conditioning Research 24.2 (2010): 370-374.‏

Li 46-48: this sounds like a very important part of the paper, but I could not understand what was done. Please, consider revising it.

We have revised this sentence as suggested:

“In the current paper, the same data that was collected from subjects performing both CPET and WAnT was used, yet the CPET data was adjusted/re- calculated in a way that represents a standard GXT (which do not include gas exchange variables).”

We have also explained it in more details at comment #12 in reviewer 1.

Li 69: minimal during the test, or minimal after the peak?

Minimal during the test.

Li 93: so you performed a maximal incremental test measuring gas exchanges, but you used the ACSM equation to predict VO2max? This does not make much sense to me.

As we aforementioned we used the age, slope and speed from the CPET data so it can represent standard exercise stress test. These variables were used to predict VO2max using ACSM equation.

Li 120: what are these values here? Are 0.93 and 0.94 correlation coefficients?

These are the correlations predicting the PP and MP from GXT using machine learning tools after applying the validation set on the equations. We have revised it in the paper and used r=0.93 and r=0.94 for PP and MP respectively.

Li 156-160: this does not make much sense to me. Which result do you get from a Wingate test that you can also get from a CPET here? This comparison is conceptually wrong, in my opinion.

We have explained this comment in details for reviewer 1 (please see aforementioned)

Shortly, it means that by integrating machine learning tools from features obtained from a single aerobic stress test, we can predict the peak and mean power outputs obtain from the Wingate anaerobic test.

Li 210: … and your conclusion is?

That even though the limitations, we have managed to provide a proof of concept on the ability to predict the mechanical power outputs using a single aerobic exercise stress test. Future research is needed to provide a generic equation that can be applied regularly when performing standard aerobic stress test.
