# Supplementary material for: A computational predictor of the anaerobic mechanical power outputs from a clinical exercise stress test
Source: PLoS One. 2023 May 5;18(5):e0283630. doi: 10.1371/journal.pone.0283630 (PMC10162510; doi:10.1371/journal.pone.0283630)
Supplement: Attachment — Submitted filename: Review_250622.docx [file pone.0283630.s004.docx]

**Review**

1. Recommendation: **Major Revision**
2. Is the manuscript technically sound, and do the data support the conclusions? **Yes**

3. Has the statistical analysis been performed appropriately and rigorously? **Yes**
4. Does the manuscript adhere to the PLOS Data Policy? **Yes**
5. Is the manuscript presented in an intelligible fashion and written in standard English? **Yes**

6. Review Comments to the Author (minimum 200 characters)

**A computational predictor of the anaerobic mechanical power outputs from a clinical exercise stress test**

**General**

**Order of authors is not consistent through submission.**

**A main issue is that you did not perform GXT but you used parameters from CPET test. CPET test require wearing a mask that may affect the exercise parameter, thus I am not convinced that it is meaningful using these adapted variables.**

**Introduction**

**Page 9, lines 4-6-** The most common standard graded exercise test (GXT) include 5 incremental increase of intensity which terminate when a heart rate (HR) of 85% to 100% of 6 age-adjusted maximal HR is achieved [5].

**The reference does not support the information in the sentence, please change reference.**

**Page 9, line 7-** Numerous studies have previously shown….

**Please provide references for this sentence.**

**Page 9, line 10-** Since measuring phosphagen and glycogen contents is complicated, these metabolic systems are usually reflected via power output measurement using the WAnT.

**Please provide references for this sentence.**

**Page 9, line 12-** Therefore, since knowing the anaerobic components is important, performing an 13 anaerobic exercise test is required.

**Why is knowing the anaerobic component is important? Please explain or provide reference. There is some explanations in line 19, but it should be clearer.**

**Page 9, line 21-** This information is of 22 special interest for athletes.

Athlete’s population is not usually performing GXT, as you pointed yourself that this test is usually performed on non- health population.

**Page 10, line 34-** Anaerobic metabolism becomes dominate and allows the generation of additional mechanical power output.

**References do not support the data in the session, please provide references that are more relevant.**

**Page 10, line 42-** outputs**…. Delete one**

**Page 11, line 63-** **References 17, 18 should be inserted after:** “which is considered as the most popular anaerobic test”.

**Page 12, line 93-** Based on this, we have extracted 25 new features representing standard GXT as detailed in supplementary Table S1.

**Please indicate the 25 features in the text.**

**Page 13, line 122- Please elaborate on the equation, for example, what Y stands for?**

**Page 13, line 122- “**Prediction of MP using features taken from CPET produced a spearman correlation 177 coefficient of r = 0.91 which is lower than the MP predictions using features taken from 178 standard GXT”

**Please explain, why is it lower in CPET?**

**Page 16, line 191- “**This feature might represent the aerobic contribution during the WAnT”.

**What feature did you mean? Also, reference 22 does not show anything about aerobic contribution….**

**Page 16, line 197- “**Therefore, this feature can give an indication of lactate accumulation following glycogen metabolism, which is used during the WAnT”.

**Again, what feature did you mean? What is the importance of this information?**

7. PLOS authors have the option to publish the peer review history of their article (https://journals.plos.org/plosone/s/editorial-and-peer-review-process#loc-peer-review-history). If published, this will include your full peer review and any attached files. Do you want your identity to be public for this peer review? **No**

7a. If you answered "Yes" above, that you would like your identity to be public, please provide your full name, as it should appear on the published peer review. Please do not sign the review on behalf of another person.

Confidential to Editor:
1. Do you have any potentially competing interests? If none, type "**None**

2. Did you receive any assistance in preparing this review? **No**

3. Do you want to get recognition for this review on Publons (https://publons.com/benefits/reviewers/how)? **No**
